# Association of Children’s Mode of School Instruction with Child and Parent Experiences and Well-Being During the COVID-19 Pandemic — COVID Experiences Survey, United States, October 8–November 13, 2020

**DOI:** 10.15585/mmwr.mm7011a1

**Published:** 2021-03-19

**Authors:** Jorge V. Verlenden, Sanjana Pampati, Catherine N. Rasberry, Nicole Liddon, Marci Hertz, Greta Kilmer, Melissa Heim Viox, Sarah Lee, Neha K. Cramer, Lisa C. Barrios, Kathleen A. Ethier

**Affiliations:** ^1^Division of Adolescent and School Health, National Center for HIV/AIDS, Viral Hepatitis, STD, and TB Prevention, CDC; ^2^CDC COVID-19 Emergency Response Team; ^3^Oak Ridge Institute for Science and Education, Oak Ridge, Tennessee; ^4^NORC at the University of Chicago, Illinois;^ 5^Division of Population Health, National Center for Chronic Disease Prevention and Health Promotion, CDC.

In March 2020, efforts to slow transmission of SARS-CoV-2, the virus that causes COVID-19, resulted in widespread closures of school buildings, shifts to virtual educational models, modifications to school-based services, and disruptions in the educational experiences of school-aged children. Changes in modes of instruction have presented psychosocial stressors to children and parents that can increase risks to mental health and well-being and might exacerbate educational and health disparities (*1*,*2*). CDC examined differences in child and parent experiences and indicators of well-being according to children’s mode of school instruction (i.e., in-person only [in-person], virtual-only [virtual], or combined virtual and in-person [combined]) using data from the COVID Experiences nationwide survey. During October 8–November 13, 2020, parents or legal guardians (parents) of children aged 5–12 years were surveyed using the NORC at the University of Chicago AmeriSpeak panel,* a probability-based panel designed to be representative of the U.S. household population. Among 1,290 respondents with a child enrolled in public or private school, 45.7% reported that their child received virtual instruction, 30.9% in-person instruction, and 23.4% combined instruction. For 11 of 17 stress and well-being indicators concerning child mental health and physical activity and parental emotional distress, findings were worse for parents of children receiving virtual or combined instruction than were those for parents of children receiving in-person instruction. Children not receiving in-person instruction and their parents might experience increased risk for negative mental, emotional, or physical health outcomes and might need additional support to mitigate pandemic effects. Community-wide actions to reduce COVID-19 incidence and support mitigation strategies in schools are critically important to support students’ return to in-person learning.

The COVID Experiences nationwide survey was administered online or via telephone during October 8–November 13, 2020 to parents of children aged 5–12 years (1,561) using NORC’s AmeriSpeak panel (*3*).^†^ A sample of adults in the AmeriSpeak panel identified as potential respondents was selected using sampling strata based on age, race/ethnicity, education, and sex of the adult. Parents with multiple children were asked to report on their child aged 5–12 years with the most recent birthday. Analyses were limited to parents of children attending a public or private school during the 2020–21 school year.^§^ On the basis of parent responses about the mode of school instruction,^¶^ three unweighted categories were constructed: in-person (434), virtual (530), and combined (326). Parents who did not select one of the prespecified modes of instruction categories or did not report their child attended a public or private school (271) were excluded from analyses. The final sample included 1,290 parents of children, 1,169 (92.9%) of whom were enrolled in public school and 121 (7.1%) enrolled in private school. Parents reported on children’s experiences and well-being, including changes since the pandemic began in physical activity and time spent outside; physical, mental, and emotional health status before and during the pandemic; and measures of current anxiety and depression.** In addition, parents reported on their own well-being and experiences, including job stability, child care challenges, and emotional distress. Unweighted frequencies or weighted prevalence estimates and 95% confidence intervals of demographic characteristics, experiences, and well-being indicators by school instruction mode were calculated. Chi-square tests identified differences by demographic characteristics. Controlling for child’s age and parent’s race/ethnicity, sex, and household income, the study calculated adjusted prevalence ratios using predicted margins in logistic regression, comparing experiences and well-being indicators by mode of instruction. P-values <0.05 were considered statistically significant. The complex sample design was accounted for using SAS-callable SUDAAN (version 11.0; RTI International). This activity was reviewed by CDC and was conducted consistent with applicable federal law and CDC policy; the study was also reviewed and approved by the Institutional Review Board of NORC at the University of Chicago.^††^

Approximately one half of parents (45.7%) reported that their child received virtual instruction, 30.9% reported in-person instruction, and 23.4% reported combined instruction (Table 1). Parents of children enrolled in public school more commonly reported that their children received virtual instruction (47.6%) compared with parents of children enrolled in private school (20.3%). Virtual instruction was also more commonly reported by Hispanic parents (65.9%), non-Hispanic other/multiracial parents (64.0%), and non-Hispanic Black parents (54.9%) than by non-Hispanic White parents (31.9%).

**TABLE 1:** Respondent, child and household characteristics, by mode of child’s school instruction* — COVID Experiences Survey,^†^ United States, October 8–November 13, 2020

Characteristic	Mode of child’s^§^ school instruction,^¶^ no., % (95% CI)								p-value^††^
	Overall		In-person only		Virtual only		Combined**		
**Total**	**1,290**	**100.0**	**434**	**30.9 (26.3–35.9)**	**530**	**45.7 (40.0–51.5)**	**326**	**23.4 (19.9–27.4)**
**Child characteristic**									
**Sex** ^§§^									0.23
Male	519	51.7 (47.1–56.3)	180	29.5 (23.9–35.7)	201	45.4 (39.2–51.8)	138	25.1 (20.2–30.7)	
Female	455	48.3 (43.7–52.9)	151	30.6 (24.0–38.1)	201	50.3 (41.1–59.4)	103	19.2 (15.1–24.0)	
**Age group, yrs**									0.03
5–8	550	41.5 (38.3–44.9)	206	35.4 (29.3–42.0)	214	45.2 (39.1–51.4)	130	19.4 (15.0–24.7)	
9–12	739	58.5 (55.1–61.7)	228	27.8 (22.7–33.4)	315	45.9 (38.8–53.1)	196	26.4 (21.5–31.9)	
**Existing emotional, mental, developmental, or behavioral condition** ^¶¶^									0.56
Yes	255	18.9 (16.0–22.1)	81	30.6 (23.3–39.1)	112	49.2 (38.8–59.7)	62	20.2 (14.8–27.0)	
No	1,032	81.1 (77.9–84.0)	352	31.0 (26.2–36.3)	417	45.0 (39.5–50.6)	263	24.0 (20.2–28.4)	
**Child’s school type**									<0.01
Public	1,169	92.9 (91.3–94.3)	352	28.3 (23.6–33.4)	507	47.6 (41.6–53.7)	310	24.1 (20.5–28.2)	
Private	121	7.1 (5.7–8.7)	82	65.6 (54.5–75.2)	23	20.3 (13.0–30.2)	16	14.2 (9.0–21.5)	
Child receives free or reduced cost lunch***									0.85
Yes	746	59.7 (56.7–62.7)	245	30.7 (26.3–35.4)	310	46.5 (40.1–53.0)	191	22.8 (18.3–28.0)	
No	541	40.3 (37.3–43.3)	189	31.4 (25.3–38.3)	218	44.6 (37.6–51.7)	134	24.0 (19.9–28.7)	
**Parent and household characteristic**									
**Sex**									0.81
Male	427	44.5 (40.8–48.3)	155	32.2 (26.2–38.9)	166	44.5 (37.3–52.0)	106	23.3 (17.6–30.1)	
Female	863	55.5 (51.7–59.2)	279	29.8 (24.4–35.9)	364	46.6 (40.1–53.2)	220	23.6 (20.4–27.0)	
**Race/Ethnicity**									<0.01
White, non-Hispanic	870	55.8 (51.3–60.3)	352	39.5 (33.6–45.7)	271	31.9 (26.4–38.0)	247	28.6 (23.7–34.0)	
Black, non-Hispanic	132	9.4 (7.3–12.1)	31	30.7 (19.8–44.1)	80	54.9 (44.9–64.5)	21	14.5 (7.3–26.6)	
Hispanic	163	23.8 (19.2–29.0)	28	17.5 (9.6–29.5)	106	65.9 (55.2–75.2)	29	16.6 (10.5–25.3)	
Other, non-Hispanic^†††^	125	11.0 (8.9–13.5)	23	16.4 (9.9–25.9)	73	64.0 (48.3–77.2)	29	19.6 (11.0–32.5)	
**Marital status**									0.39
Married or living with partner	1,050	82.5 (79.7–85.0)	366	30.9 (26.1–36.3)	429	46.6 (40.3–53.0)	255	22.5 (18.7–26.8)	
Never married, divorced, widowed, or separated	240	17.5 (15.0–20.3)	68	30.6 (24.2–37.8)	101	41.5 (33.7–49.7)	71	27.9 (21.1–35.9)	
**Parental education**									0.29
Less than high school or high school graduate	203	31.2 (27.0–35.8)	71	33.6 (25.8–42.4)	82	45.9 (35.5–56.6)	50	20.5 (14.4–28.5)	
Some college or technical school or associate degree	493	26.3 (23.6–29.2)	166	31.8 (25.2–39.2)	201	43.1 (36.9–49.4)	126	25.2 (19.9–31.3)	
Bachelor’s degree or higher	594	42.5 (38.7–46.3)	197	28.3 (23.3–33.9)	247	47.2 (41.1–53.3)	150	24.5 (20.7–28.8)	
**Annual household income**									0.56
≤$34,999	279	26.3 (22.9–30.0)	82	33.0 (25.2–41.9)	123	48.5 (38.4–58.6)	74	18.5 (13.4–25.1)	
$35,000–$49,999	157	13.6 (11.2–16.3)	51	27.2 (18.1–38.7)	64	50.1 (37.3–62.9)	42	22.7 (14.4–33.8)	
$50,000–$74,999	266	17.4 (15.2–19.9)	89	32.8 (26.5–39.8)	110	42.2 (32.9–52.0)	67	25.0 (18.4–33.1)	
$75,000–$99,999	228	14.6 (12.4–17.1)	87	31.4 (24.5–39.3)	90	42.5 (34.9–50.5)	51	26.1 (18.0–36.2)	
≥$100,000	360	28.2 (24.7–31.8)	125	29.2 (22.7–36.7)	143	44.8 (37.0–52.8)	92	26.0 (20.9–31.9)	

**Abbreviation:** CI = confidence interval.

* Table shows unweighted frequencies, weighted overall and row percentages, and weighted 95% CIs.

^†^ https://amerispeak.norc.org/Documents/Research/AmeriSpeak%20Technical%20Overview%202019%2002%2018.pdf

^§^ Sampled parents with multiple children were asked to report on their child aged 5–12 years with the most recent birthday.

^¶^ Among those who responded that their child attended a public or private school in the 2020–21 school year, mode of instruction categories are based on response to the question “During the current school year (2020/21), how has [the child] attended school? Select all that apply.” Possible responses were “in-person full time,” “virtual/online full-time,” “in-person part-time and virtual part-time (meaning in school several days a week or several weeks each month, and virtual learning the other days/weeks),” or “other, please specify.” Three mutually exclusive categories were based on the selection of: 1) only in-person full time; 2) only virtual/online full-time; or 3) combination of in-person full time, virtual/online full-time, or in-person part-time and virtual part-time.

** Indicates a combination of in-person and virtual instruction.

^††^ Chi-square test was used to identify overall differences in child and parent demographics and household characteristics by mode of school instruction.

^§§^ First name-based imputation was used to impute sex for 148 children who were missing information on sex. After imputation, child’s sex remained missing for 316 records (24.5%).

^¶¶^ Any emotional, mental, developmental, or behavioral condition for which the child needed or received treatment, therapy, or counseling. Examples include anxiety, depression, attention deficit disorder or attention deficit hyperactivity disorder, autism spectrum disorder, or intellectual disability.

*** Question assessed whether child ever received free or reduced-cost school meals (i.e., breakfast, lunch, or both).

^†††^ Includes other non-Hispanic races and non-Hispanic multiracial persons.

Parents of children receiving virtual instruction were more likely than were parents of children receiving in-person instruction to report that their children experienced decreased physical activity (62.9% versus 30.3%), time spent outside (58.0% versus 27.4%), in-person time with friends (86.2% versus 69.5%), virtual time with friends (24.3% versus 12.6%), and worsened mental or emotional health (24.9% versus 15.9%) (Table 2). Parents of children receiving combined instruction were also more likely than were those of children receiving in-person instruction to report that their children experienced decreased physical activity (52.1% versus 30.3%), time spent outside (42.4% versus 27.4%), in-person time with friends (84.1% versus 69.5%), and worsened mental or emotional health (24.7% versus 15.9%). Parents of children receiving virtual instruction were more likely than were parents of children receiving combined instruction to report that their children experienced decreased physical activity (62.9% versus 52.1%) and time spent outside (58.0% versus 42.4%). 

**TABLE 2:** Weighted prevalence (%) and adjusted prevalence ratios (aPRs) of parent report of child experiences and well-being indicators, by mode of child’s school instruction* — COVID Experiences Survey,**^†^** United States, October 8–November 13, 2020

Characteristic	Mode of child’s^§^ school instruction,^¶^ % (95% CI)				Adjusted comparisons for child experiences and well-being by mode of child’s school instruction, aPR** (95% CI)		
	Overall (N = 1,290)	In-person only (n = 434)	Virtual only (n = 530)	Combined^††^ (n = 326)	Virtual only versus in-person only	Combined versus in-person only	Virtual only versus combined
**Child experience**							
**Change in physical activity** ^§§^							
Decreased	50.3 (46.5–54.0)	30.3 (25.1–36.1)	62.9 (58.1–67.4)	52.1 (45.8–58.3)	1.9 (1.6–2.3)^¶¶^	1.6 (1.3–1.9)^¶¶^	1.2 (1.0–1.4)^¶¶^
No impact or increased	49.7 (46.0–53.5)	69.7 (63.9–74.9)	37.1 (32.6–41.9)	47.9 (41.7–54.2)	—	—	—
**Change in spending time outside** ^§§^							
Decreased	44.9 (40.9–48.9)	27.4 (21.9–33.8)	58.0 (52.2–63.5)	42.4 (36.1–49.0)	1.8 (1.4–2.2)^¶¶^	1.4 (1.1–1.8)^¶¶^	1.3 (1.1–1.6)^¶¶^
No impact or increased	55.1 (51.1–59.1)	72.6 (66.2–78.1)	42.0 (36.5–47.8)	57.6 (51.0–63.9)	—	—	—
**Change in spending time with friends in-person** ^§§^							
Decreased	80.5 (76.9–83.7)	69.5 (62.7–75.5)	86.2 (81.4–89.9)	84.1 (76.3–89.6)	1.2 (1.1–1.3)^¶¶^	1.2 (1.1–1.3)^¶¶^	1.1 (0.9–1.2)
No impact or increased	19.5 (16.3–23.1)	30.5 (24.5–37.3)	13.8 (10.1–18.6)	15.9 (10.4–23.7)	—	—	—
**Change in spending time with friends virtually for non-educational purposes** ^§§^							
Decreased	18.6 (15.6–22.0)	12.6 (8.6–18.2)	24.3 (19.1–30.4)	15.3 (10.6–21.5)	1.7 (1.1–2.7)^¶¶^	1.2 (0.8–2.0)	1.4 (0.9–2.1)
No impact or increased	81.4 (78.0–84.4)	87.4 (81.8–91.4)	75.7 (69.6–80.9)	84.7 (78.5–89.4)	—	—	—
**Child well-being**							
Change in physical health***							
Worse	12.6 (10.2–15.6)	9.3 (6.2–13.6)	14.7 (10.3–20.5)	13.0 (9.4–17.8)	1.4 (0.8–2.3)	1.3 (0.8–2.2)	1.1 (0.7–1.7)
Better or no change	87.4 (84.4–89.8)	90.7 (86.4–93.8)	85.3 (79.5–89.7)	87.0 (82.2–90.6)	—	—	—
**Change in mental or emotional health** ^†††^							
Worse	22.1 (19.8–24.7)	15.9 (12.5–20.1)	24.9 (21.4–28.8)	24.7 (20.4–29.5)	1.6 (1.2–2.2)^¶¶^	1.5 (1.1–2.0)^¶¶^	1.1 (0.9–1.4)
Better or no change	77.9 (75.3–80.2)	84.1 (79.9–87.5)	75.1 (71.2–78.6)	75.3 (70.5–79.6)	—	—	—
**Depression** ^§§§^							
With elevated symptoms	4.4 (2.8–6.9)	3.6 (1.9–6.9)	5.3 (2.7–10.3)	3.7 (1.8–7.3)	1.4 (0.6–3.1)	1.0 (0.4–2.5)	1.4 (0.6–3.3)
Without elevated symptoms	95.6 (93.1–97.2)	96.4 (93.1–98.1)	94.7 (89.7–97.3)	96.3 (92.7–98.2)	—	—	—
**Anxiety** ^§§§^							
With elevated symptoms	6.3 (5.0–7.8)	6.7 (4.4–10.1)	7.0 (5.1–9.5)	4.4 (2.5–7.6)	1.1 (0.6–2.0)	0.7 (0.3–1.4)	1.6 (0.8–3.2)
Without elevated symptoms	93.7 (92.2–95.0)	93.3 (89.9–95.6)	93.0 (90.5–94.9)	95.6 (92.4–97.5)	—	—	—
**Psychological stress** ^§§§^							
With elevated symptoms	9.2 (7.3–11.5)	9.5 (6.7–13.4)	9.2 (6.2–13.3)	8.7 (6.2–12.0)	1.0 (0.6–1.7)	0.9 (0.6–1.4)	1.2 (0.7–1.9)
Without elevated symptoms	90.8 (88.5–92.7)	90.5 (86.6–93.3)	90.8 (86.7–93.8)	91.3 (88.0–93.8)	—	—	—

**Abbreviation:** CI = confidence interval.

* Table shows weighted overall and column percentages and corresponding 95% CIs, and adjusted prevalence ratios and 95% CIs.

^†^ https://amerispeak.norc.org/Documents/Research/AmeriSpeak%20Technical%20Overview%202019%2002%2018.pdf

^§^ Sampled parents with multiple children were asked to report on their child aged 5–12 years with the most recent birthday.

^¶^ Among those who responded that their child attended a public or private school in the 2020–21 school year, mode of instruction categories are based on response to the question “During the current school year (2020/21), how has [the child] attended school? Select all that apply.” Possible responses were “in-person full time,” “virtual/online full-time,” “in-person part-time and virtual part-time (meaning in school several days a week or several weeks each month, and virtual learning the other days/weeks),” or “other, please specify.” Three mutually exclusive categories were based on the selection of: 1) only in-person full time; 2) only virtual/online full-time; or 3) combination of in-person full time, virtual/online full-time, or in-person part-time and virtual part-time.

** aPR adjusted for parent’s race/ethnicity and sex, household income, and child’s age. aPR was not adjusted for all child characteristics (sex; existing emotional, mental, developmental, or behavioral condition; school type; receipt of free or reduced-cost lunch) and parent characteristics (marital status or education).

^††^ Indicates a combination of in-person and virtual instruction.

^§§^ Question assessed how the COVID-19 pandemic has affected each behavior or experience.

^¶¶^ p-values <0.05 were considered statistically significant. Some 95% CIs include 1.0 because of rounding.

*** Question items asked parents to rate child’s physical health (very good, good, fair, or poor) before the COVID-19 pandemic (February 2020) and current physical health. Any decline in physical health was categorized as “worse” and any improvement or no change in physical health was categorized as “better or no change.”

^†††^ Question items asked parents to rate the child’s mental and emotional health (very good, good, fair, or poor) before the COVID-19 pandemic (February 2020) and current mental or emotional health. Any decline in mental or emotional health was categorized as “worse” and any improvement or no change in mental or emotional health was categorized as “better or no change.”

^§§§^ Patient Reported Outcomes Measurement Information System (http://www.healthmeasures.net/) parent proxy report scales short forms, depressive symptoms, anxiety symptoms, and psychological stress. Raw scores are converted to T-scores, with a mean of 50 and standard deviation (SD) of 10 referenced to a healthy cohort. High scores indicate more of the concept measured. Elevated symptoms of depression (moderately severe/severe), anxiety (moderately severe/severe), and psychological stress (moderately high/very high) include those with T-scores≥65, 1.5 SDs higher than the mean of the reference population. Automated scoring was provided through Northwestern University, HealthMeasures. https://www.assessmentcenter.net/ac_scoringservice

Parents of children receiving virtual instruction were also more likely than were parents of children receiving in-person instruction to report loss of work^§§^ (42.7% versus 30.6%), job stability concerns (26.6% versus 15.2%), child care challenges (13.5% versus 6.8%), conflict between working and providing child care (14.6% versus 8.3%), emotional distress (54.0% versus 38.4%), and difficulty sleeping (21.6% versus 12.9%) (Table 3). Parents of children receiving combined instruction were more likely than were those of children receiving in-person instruction to report loss of work (40.1% versus 30.6%) and conflict between working and providing child care (14.2% versus 8.3%). Parents of children receiving virtual instruction were more likely than were parents of children receiving combined instruction to report experiencing emotional distress (54.0% versus 42.9%).

**TABLE 3:** Weighted prevalence (%) and adjusted prevalence ratios (aPRs) of parent experiences and well-being indicators, by mode of child’s school instruction* — COVID Experiences Survey,^†^ United States, October 8–November 13, 2020

Characteristic	Mode of child’s school instruction,^§^ % (95% CI)				Adjusted comparisons for parent experiences and well-being by mode of child’s school instruction, aPR^¶^ (95% CI)		
	Overall (N = 1,290)	In-person only (n = 434)	Virtual only (n = 530)	Combined** (n = 326)	Virtual only versus in-person only	Combined** versus in-person only	Virtual only versus combined**
**Parent experience**							
**Loss of work^††^**							
Yes	38.3 (34.5–42.3)	30.6 (25.4–36.3)	42.7 (36.5–49.1)	40.1 (31.9–48.8)	1.4 (1.1–1.8)^§§^	1.4 (1.0–1.8)^§§^	1.0 (0.8–1.3)
No	61.7 (57.7–65.5)	69.4 (63.7–74.6)	57.3 (50.9–63.5)	59.9 (51.2–68.1)	—	—	—
**Concern about job stability** ^¶¶^							
Often	21.5 (18.2–25.1)	15.2 (12.0–19.2)	26.6 (21.5–32.4)	19.6 (14.1–26.5)	1.6 (1.3–2.1)^§§^	1.3 (0.9–1.9)	1.2 (0.8–1.8)
Sometimes or never	78.5 (74.9–81.8)	84.8 (80.8–88.0)	73.4 (67.6–78.5)	80.4 (73.5–85.9)	—	—	—
**Child care challenges** ^¶¶^							
Often	10.5 (8.6–12.7)	6.8 (4.5–10.3)	13.5 (10.3–17.4)	9.5 (6.5–13.7)	1.7 (1.1–2.7)^§§^	1.4 (0.9–2.2)	1.2 (0.7–2.0)
Sometimes or never	89.5 (87.3–91.4)	93.2 (89.7–95.5)	86.5 (82.6–89.7)	90.5 (86.3–93.5)	—	—	—
**Conflict between working and providing child care** ^¶¶^							
Often	12.6 (10.5–14.9)	8.3 (5.9–11.5)	14.6 (11.7–18.1)	14.2 (10.0–19.7)	1.5 (1.0–2.3)^§§^	1.7 (1.1–2.5)^§§^	0.9 (0.6–1.5)
Sometimes or never	87.4 (85.1–89.5)	91.7 (88.5–94.1)	85.4 (81.9–88.3)	85.8 (80.3–90.0)	—	—	—
Increased substance use***							
Yes	16.5 (13.8–19.6)	13.7 (10.5–17.8)	16.4 (12.0–21.9)	20.5 (15.1–27.1)	1.2 (0.8–1.7)	1.5 (1.0–2.3)	0.8 (0.5–1.1)
No	83.5 (80.4–86.2)	86.3 (82.2–89.5)	83.6 (78.1–88.0)	79.5 (72.9–84.9)	—	—	—
**Parent well-being**							
**Emotional Distress** ^†††^							
A lot or moderate	46.6 (43.3–49.9)	38.4 (32.7–44.5)	54.0 (48.8–59.1)	42.9 (35.9–50.1)	1.4 (1.2–1.6)^§§^	1.1 (0.9–1.4)	1.2 (1.1–1.5)^§§^
Little or no	53.4 (50.1–56.7)	61.6 (55.5–67.3)	46.0 (40.9–51.2)	57.1 (49.9–64.1)	—	—	—
**Difficulty managing emotions** ^¶¶^							
Often	13.5 (11.1–16.3)	11.0 (7.8–15.2)	14.3 (11.0–18.5)	15.2 (10.5–21.5)	1.1 (0.7–1.7)	1.4 (0.9–2.0)	0.8 (0.5–1.2)
Sometimes or never	86.5 (83.7–88.9)	89.0 (84.8–92.2)	85.7 (81.5–89.0)	84.8 (78.5–89.5)	—	—	—
**Difficulty sleeping or insomnia** ^¶¶^							
Often	17.7 (15.3–20.5)	12.9 (9.8–16.8)	21.6 (17.8–26.1)	16.4 (11.8–22.5)	1.6 (1.2–2.2)^§§^	1.2 (0.9–1.7)	1.3 (0.9–1.8)
Sometimes or never	82.3 (79.5–84.7)	87.1 (83.2–90.2)	78.4 (73.9–82.2)	83.6 (77.5–88.2)	—	—	—

**Abbreviation:** CI = confidence interval.

* Table shows weighted overall and column percentages and corresponding 95% CIs, and adjusted prevalence ratios and 95% confidence intervals.

^†^ https://amerispeak.norc.org/Documents/Research/AmeriSpeak%20Technical%20Overview%202019%2002%2018.pdf

^§^ Among those who responded that their child attended a public or private school in the 2020–21 school year, mode of instruction categories are based on response to the question “During the current school year (2020/21), how has [the child] attended school? Select all that apply.” Possible responses were “in-person full time,” “virtual/online full-time,” “in-person part-time and virtual part-time (meaning in school several days a week and virtual learning the other days/weeks),” or “other, please specify.” Three mutually exclusive categories were based on the selection of: 1) only in-person full time; 2) only virtual/online full-time; or 3) combination of in-person full time, virtual/online full-time, or in-person part-time and virtual part-time.

^¶^ aPR adjusted for parent’s race/ethnicity and sex, household income, and child’s age. aPR was not adjusted for all child characteristics (sex; existing emotional, mental, developmental, or behavioral condition; school type; receipt of free or reduced-cost lunch) and parent characteristics (marital status or education).

** Indicates a combination of in-person and virtual instruction.

^††^ Question assessed whether the respondent experienced or was experiencing any of the following as a result of the pandemic: loss of work, decreased hours or wages, furloughed, or laid off.

^§§^ p-values <0.05 were considered statistically significant. Some 95% CIs include 1.0 because of rounding.

^¶¶^ Question assessed how frequently the respondent experienced the following since the COVID-19 pandemic began: concern about job stability, child care challenges, conflict between working and providing child care, difficulty managing emotions, difficulty sleeping, or insomnia.

*** Question assessed whether the respondent started or increased using substances to help cope with stress or emotions during the COVID-19 pandemic. Substance use includes alcohol, legal or illegal drugs, or prescription drugs that are taken in a way not recommended by a doctor.

^†††^ Question assessed how much emotional distress such as increased sadness, anxiety, and worry the respondent experienced related to the COVID-19 pandemic.

## Discussion

Findings from this survey of parents of children aged 5–12 years indicate that parents whose children received virtual or combined instruction were more likely to report higher prevalence of risk on 11 of 17 indicators of child and parental well-being than were parents whose children received in-person instruction. Among nine examined indicators of children’s well-being, five differed significantly by the instruction mode that children received. These differences reflected higher prevalences of negative indicators of well-being for children receiving virtual or combined instruction than for children receiving in-person instruction. Parents of children receiving virtual or combined instruction more frequently reported that their child’s mental or emotional health worsened during the pandemic and that their time spent outside, in-person with friends, and engaged in physical activity decreased. Regular physical activity is associated with children’s improved cardiorespiratory fitness, increased muscle and bone strength, and reduced risk for depression, anxiety, and chronic health conditions (e.g., diabetes); therefore, these differences in physical activity are concerning (*4*,*5*). Likewise, isolation and limited physical and outside activity can adversely affect children’s mental health (*6*).

Among the eight examined indicators of parental well-being, six differed significantly by mode of instruction received by the children. Parents of children receiving virtual instruction more frequently reported their own emotional distress, difficulty sleeping, loss of work, concern about job stability, child care challenges, and conflict between working and providing child care than did parents whose children were receiving in-person instruction. Parents of children receiving combined instruction also reported conflict between working and providing child care and loss of work more often than did parents of children receiving in-person instruction. Chronic stress can negatively affect physical and mental health of both children and parents, especially without social and economic supports, and could contribute to widening of educational and health disparities (*2*,*3*,*7*,*8*). In this study, Black, Hispanic, and non-Hispanic other or multiracial parents were more likely than White parents to report children receiving virtual instruction. Further research is needed to understand whether virtual instruction has disproportionately negative impacts on child and parent health outcomes among racial and ethnic minorities and communities disproportionately affected by COVID-19. The role of other contextual and interpersonal factors on experiences of stress and risks to well-being in relation to the pandemic needs further exploration.

Schools are central to supporting children and families, providing not only education, but also opportunities to engage in activities to support healthy development and access to social, mental health, and physical health services, which can buffer stress and mitigate negative outcomes. However, the pandemic is disrupting many school-based services, increasing parental responsibilities and stress, and potentially affecting long-term health outcomes for parents and children alike, especially among families at risk for negative health outcomes from social and environmental factors (*2*,*7*,*9*,*10*). These findings suggest that virtual instruction might present more risks than does in-person instruction related to child and parental mental and emotional health and some health-supporting behaviors, such as engaging in physical activity, with combined instruction falling between.

The findings in this report are subject to at least six limitations. First, responses from this incentivized, English-language survey might not represent the broader U.S. population, and the limited sample size and response rate might affect generalizability. Second, although survey responses were weighted to approximate representativeness of U.S. household demographics, findings might not be representative of all U.S. students and children aged 5–12 years. Third, parent self-reports and proxy reports for children are subject to social desirability, proxy-response, and recall biases. Fourth, parents of children receiving combined instruction did not provide details on how often children received in-person or virtual instruction; additional variation within this category might exist. Fifth, the study did not adjust for all potential confounders such as community COVID-19 transmission levels and some household and individual characteristics (e.g., urbanicity or rurality, or number of children in the household). Finally, causality between instruction mode and examined indicators of well-being cannot be inferred from this cross-sectional study.

Parents of children receiving in-person instruction reported the lowest prevalence of negative indicators of child and parental well-being. Children receiving virtual or combined instruction and their parents might need additional support to mitigate stress, including linkage to social and mental health services and opportunities to engage in safe physical activity to reduce risks associated with chronic health conditions. Culturally applicable support programming and resources might be warranted to meet community needs, ensure equitable access to services, and address health or educational inequities for families from racial and ethnic minority groups. These findings highlight the importance of in-person learning for children’s physical and mental well-being and for parents’ emotional well-being. Community-wide actions^¶¶^ to reduce COVID-19 incidence and support mitigation strategies in schools*** are critically important to support students’ return to in-person learning.

**Summary**

**What is already known about the topic?**

COVID-19–associated schooling changes present stressors to children and parents that might increase risks to mental health and well-being.

**What is added by this report?**

In a probability-based survey of parents of children aged 5–12 years, 45.7% reported that their children received virtual instruction only, 30.9% in-person only, and 23.4% combined virtual and in-person instruction. Findings suggest that virtual instruction might present more risks than does in-person instruction related to child and parental mental and emotional health and some health-supporting behaviors.

**What are the implications for public health practice?**

Children not receiving full-time, in-person instruction and their parents might need additional supports to mitigate pandemic impacts.

## Footnotes

* The AmeriSpeak panel includes approximately 40,000 households recruited using random sampling from an address-based sample with mail, e-mail, Internet, telephone, and in-person follow-up. https://amerispeak.norc.org/Documents/Research/AmeriSpeak%20Technical%20Overview%202019%2002%2018.pdf

† Among persons sampled, 32.9% completed a screener to determine eligibility; among those eligible, the survey completion rate was 97.4%. AmeriSpeak panel members receive modest incentives in the form of “AmeriPoints” for participation in surveys.

§ Question asked was “Is [the child] enrolled in any of the following for the 2020/21 school year?” Possible responses were “public school,” “private school,” “homeschool,” or “is not enrolled in any school.” Only respondents selecting public or private school were included.

¶ Among those who responded that their child attended a public or private school in the 2020–21 school year, mode of instruction categories were based on response to the question “During the current school year (2020/21), how has [the child] attended school? Select all that apply.” Possible responses were “in-person full time,” “virtual/online full-time,” “in-person part-time and virtual part-time (meaning in school several days a week or several weeks each month, and virtual learning the other days/weeks),” or “other, please specify.” Three mutually exclusive categories were based on the selection of 1) only in-person full time; 2) only virtual/online full-time; or 3) combination of in-person full time, virtual/online full-time, or in-person part-time and virtual part-time.

** Patient Reported Outcomes Measurement Information System (http://www.healthmeasures.net/) parent proxy report scales short forms, depressive symptoms, anxiety symptoms, and psychological stress. Raw scores are converted to T-scores, with a mean of 50 and standard deviation (SD) of 10 referenced to a healthy cohort. High scores indicate more of the concept measured. The reported elevated symptoms of depression (moderately severe/severe), anxiety (moderately severe/severe), and psychological stress (moderately high/very high) include those with T-scores ≥65, 1.5 SDs higher than the mean of the reference population. Automated scoring was provided through Northwestern University, HealthMeasures. https://www.assessmentcenter.net/ac_scoringservice

†† 45 C.F.R. part 46; 21 C.F.R. part 56; 42 U.S.C. Sect. 241(d), 5 U.S.C. Sect. 552a, 44 U.S.C. Sect. 3501 et seq. 45 C.F.R. part 46; 21 C.F.R. part 56.

§§ Question assessed whether the parent experienced or was experiencing any of the following as a result of the COVID-19 pandemic: loss of work, decreased hours or wages, furloughed, or laid off.

¶¶
https://www.cdc.gov/coronavirus/2019-ncov/community/community-mitigation.html

***
https://www.cdc.gov/coronavirus/2019-ncov/community/schools-childcare/operation-strategy.html
